# Molecular docking analysis of alkaloid compounds with beta-catenin towards the treatment of colon cancer

**DOI:** 10.6026/97320630016283

**Published:** 2020-03-31

**Authors:** Rajagopal Ponnulakshmi, Veeraraghavan Vishnupriya, Surapaneni Krishna Mohan, Srinivasan Abilasha, Govindan Ramajayam, Periyasamy Vijayalakshmi, Manikkam Rajalakshmi, Jayaraman Selvaraj

**Affiliations:** 1 Central Research Laboratory, Meenakshi Academy of Higher Education and Research (Deemed to be University), Chennai-600 078, India; 2 Department of Biochemistry, Saveetha Dental College and Hospitals, Saveetha Institute of Medical and Technical Sciences, Saveetha University, Chennai - 600 077, India; 3 Department of Biochemistry, Panimalar Medical College Hospital and Research Institute, Varadharajapuram, Poonamallee, Chennai-600 123, Chennai, Tamil Nadu, India; 4 Department of Anatomy, Asan Memorial Dental College and Hospital, Asan Nagar, Chengalpattu, Tamil Nadu, India; 5 Multi Disciplinary Research Unit, Madurai Medical College, TamilNadu, India; 6 DBT-BIF Centre, PG and Research Department of Biotechnology & Bioinformatics, Holy Cross College (autonomous), Trichy, Tamil Nadu, India

**Keywords:** Colon cancer, β-catenin, Molecular docking, ADME

## Abstract

It is known that beta-catenin is associated with fibromatosis, sarcoma and mesenchymal tumor. Therefore, it is of interest to design an effective inhibtitor to the target protein beta-catenin. In this study, we report the molecular docking analysis of alkaloid compounds (aristolochicacid, cryptopleurine, demecolcine, fagaronine and thalicarpine) with beta-catenin for further consideration towards the design and development of potential inhintors for the treatmnet of colon cancer.

## Background

The WNT/β-catenin pathway is implicated in many forms of human disease including cancer β-catenin, a key factor in the Wnt signaling involved in the tumorigenesis of colorectal cancer. The high expression and dysregulation of β-catenin, which is related to the Wnt signaling pathway, is involved in various human diseases, especially colon cancers [1,2].β-Catenin, a messenger molecule relevant to growth and survival is degraded through the ubiquitin-proteasome pathway. Recently, evidence has also indicated that the dysregulation of Wnt/β-catenin signaling has been implicated in hematological malignancies, including MM [3].The related factors include increased expression of Wnt transcriptional cofactors and associated micro RNAs and disturbed epigenetics and posttranslational modification processes [4,5]. WNT/β-catenin signaling is also an evolutionarily conserved pathway that plays a crucial role in cellular proliferation, differentiation, and migration in multiple organ systems [6]. WNT activation can induce two different pathways, the β-catenin-dependent canonical pathway and the β-catenin-independent non-canonical pathway. In the absence of WNT ligands, β-catenin is recruited and degraded by the destruction complex. Binding of WNT to its receptors disrupts the destruction complex, thereby inducing cytoplasmic accumulation of β-catenin and subsequent translocation to the nucleus [7]. Over 90% of colorectal cancer is carried out by somatic mutations in Wnt signalling constituent genes like adenomatous polyposis coli (APC) tumor suppressor gene and β-catenin genes, resulting in constitutive activation of Wnt signalling [8]. This in turn guides to the generation of colon cancer, which are intrinsically resistant to conventional chemotherapy. Genetic changes in the adenomatous polyposis coli (APC) gene have been recognized in familial adenomatous polyposis coli and determined in a group of sporadic colorectal cancers [9]. Hence, therapeutics that can block Wnt signaling are possible to get rid of cancer cells and treat the disease. Therefore, discovery of new genes involved or deregulated in CRCs may give vital insights for development of new sustainable therapeutic interventions. In order to design new inhibitor that deregulate the β-catenin activity ten alkaloid natural compounds were selected for the present study (Table 1).

## Materials and Methods:

### Protein preparation:

**Figure 1:**
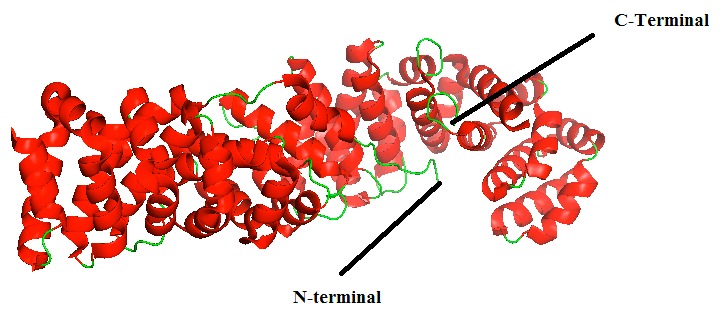
Structure of the beta-catenin

High–resolution structure of Beta-catenin (Figure 1) was downloaded from PDB (PDB Id: 1JDH) [10]. In the first of protein preparations removing other chains expect A chain and then water molecules which was observed crystallographic ally was removed. Finally prepared protein was uploaded to the Patch dock server for docking studies.

### Ligand preparation:

In our present study, selected ten natural alkaloid compounds were retrieved from pubchem database in SDF format and it was converted as PDB file format using Online Smile Translator. Energy minimizations of ligands were performed using by ChemBio 3D Ultra 12.0, based on the reported method. Energy minimized structures of ligands were uploaded in patch dock for docking analysis.

### Molecular docking:

The Patchdock server was used to find out the interaction between βcantenine and selected natural alkaloid compounds. PatchDock identified the top first candidate solutions based on shape complementarily of soft molecular surfaces. The clustering RMSD was set to 4.0 Å as proposed by the software developer for bigger molecules and the complex type was set to default. The PatchDock algorithm splits the Connolly dot surface illustration of the molecules into concave, convex and flat patches. Then, complementary patches are coordinated in order to create the candidate transformations of docked complex (the candidate transformations are the docked complexes of specified receptor and ligand molecule based on the patchdock theory). The result was retrieved from the e-mail address given and downloaded [11,12].

### Molecular descriptors calculation:

Smiles notation of compounds was used to calculate the molecular descriptors of selected compounds using Molinspiration (www.molinspiration.com). They molecular descriptors like log P, molecular weight, polar surface area, number of atoms, number of rotatable bond, number of O or N, number of OH or NH, ion channel modulator, drug-likeness and number of violations to Lipinski's rule were calculated in the present study [13].

### Analysis of ADME of selected compounds:

The ADME calculation of selected natural compounds was performed by Lipinski filter (http://www.scfbio-iitd.res.in/software/drugdesign/lipinski.jsp), according to which an orally active drug must obey at least of four of the five laid down criterion for drug likeness namely: molecular mass, cLogP, hydrogen donor and acceptor and molar refractive index [14].

## Results and discussion:

**Figure 2:**
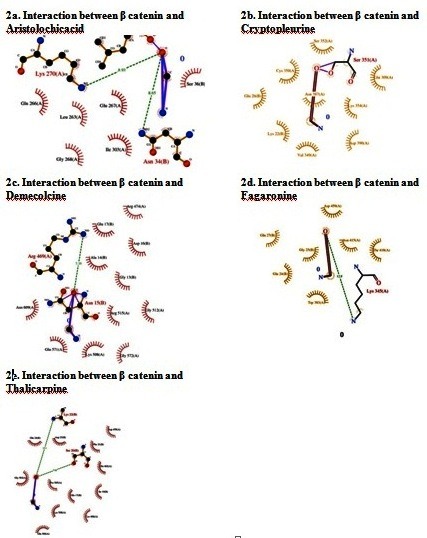
Missing: Ligplot analysis of docked complex; (a) Interaction between β catenin and Aristolochicacid (b) Interaction between β catenin and Cryptopleurine; (c) Interaction between β catenin and Demecolcine; (d) Interaction between β catenin and Fagaronine; (e) Interaction between β catenin and Thalicarpine

We took 10 alkaloid groups of natural compounds for the study. Based on the analysis of docking studies best five were shown in Table 2. The atomic contact energy (ACE) value of selected 5-alkaloid compounds range from -191.07 to -131.13 Kcal/. Thus from the calculated ACE values it can be inferred that these compounds showed the favorable binding energy with β-catenin protein. Ligplot analysis shows the hydrogen bonding and the length of their interaction with the key residues of the beta-catenin in the active site pocket. Among the residues which play a vital role in the mechanisms of action we found that the SER 20, ASN 34, LYS 22, LYS 270, SER 351, ARG 469, LYS 345 are the main interacting amino acids residues (Figure 2). With regard to physiochemical properties, all the five alkaloid compounds showed nil violation and complied well with the Lipinski's rule of five as shown in Table 3. In the same way, Table 4 shows the ADME profile of the five selected alkaloids; all the ligands are predicted to have high gastrointestinal (GI) absorption effect. Thus the results of PatchDock and ADME analysis clearly showed that selected five alkaloids group of natural compounds have the capacity to inhibit the β-catenin protein.

## Conclusions:

We report the binding properties of phytocompounds like Aristolochicacid, Cryptopleurine, Demecolcine, Fagaronine, Thalicarpine with beta-catenin in the context of colorectal cancer for further consideration and evaluation towards potential treatment.

## Figures and Tables

**Table 1:** List of selected alkaloids compounds

S. No	Compound Name	Source of plant
1	Aristolochic acid	Aristolochia indica L.
2	Camptothecin	Camptotheca acuminata Decne
3	Colchicine	Colchicum speciosum Steven
4	Cryptopleurine	Crinum macrantherium Engl
5	Epipodophyllotoxin	Podophyllum species L.
6	Demecolcine	Colchicumspeciosum Steven
7	Fagaronine	Fagara zanthoxyloides Lam
8	Oxyacanthine	Berberis asiatica Roxb.
9	Thalicarpine	Thalictrum dasycarpum Fisch

**Table 2:** Molecular docking results obtained through Patch dock server

S. No	Compound name	Score	ACE
1	Aristolochicacid	3972	-133.63
2	Cryptopleurine	4662	-191.07
3	Demecolcine.	4392	-131.13
4	Fagaronine	4278	-133.21
5	Thalicarpine	6476	-172.51

**Table 3:** Calculated molecular Descriptors using Mol inspiration

Compound name	Mi LogP^a^	TP Sa^b^	N atoms^c^	MW ^d^	N On^e^	N OHNH^f^	N Violat ions^g^	nrotb^h^	volume^i^
Aristolochicacid	3.57	110.8	25	341.3	8	1	0	3	271.8
Cryptopleurine	4.55	30.9	28	377.4	4	0	0	3	357.4
Demecolcine	1.74	66	27	371.4	6	1	0	3	357.4
Fagaronine	0	51.8	26	350.4	5	1	0	3	316.3
^a^Logarithm of partition coefficient between n-octanol and water (miLogP).^b^Topological polar surface area (TPSA).^c^Number of hydrogen bond acceptors (n-ON). ^d^Number of hydrogen bond donors (n-OHNH).^e^Number of rotatable bonds (n-rotb).^f^Percentage of absorption (%ABS).^g^Molecular weight (MW)

**Table 4:** Calculated ADME Properties

Compound name	Molecular Mass ^a^	Hydrogen bond donor ^b ^	Hydrogen bond donor ^c ^	LOGP ^d ^	Molar Refractivity ^e ^
Aristolochicacid	341	1	7	3.336698	87.742668
Cryptopleurine	377	0	4	4.696699	111.929962
Demecolcine.	371	1	6	2.6694	103.425674
Fagaronine	350	1	4	3.341899	99.786171
Thalicarpine	696	0	10	5.30418	68.041779
^a^Molecular mass less than 500 Dalton; ^b^High lipophilicity (expressed as LogP less than 5); ^c^Less than 5 hydrogen bond donors; ^d^Less than 10 hydrogen bond acceptors;^e^Molar refractivity should be between 40-130.
